# Boosting exciton mobility approaching Mott-Ioffe-Regel limit in Ruddlesden−Popper perovskites by anchoring the organic cation

**DOI:** 10.1038/s41467-024-45740-y

**Published:** 2024-02-29

**Authors:** Yiyang Gong, Shuai Yue, Yin Liang, Wenna Du, Tieyuan Bian, Chuanxiu Jiang, Xiaotian Bao, Shuai Zhang, Mingzhu Long, Guofu Zhou, Jun Yin, Shibin Deng, Qing Zhang, Bo Wu, Xinfeng Liu

**Affiliations:** 1 South China Academy of Advanced Optoelectronics, South China Normal University, Guangzhou, 510006 P.R. China; 2 CAS Key Laboratory of Standardization and Measurement for Nanotechnology, National Center for Nanoscience and Technology, Beijing, 100190 P.R. China; 3 University of Chinese Academy of Sciences, Beijing, 100049 P.R. China; 4 School of Materials Science and Engineering, Peking University, Beijing, 100871 P.R. China; 5 Department of Applied Physics, The Hong Kong Polytechnic University, Hung Hom, Kowloon, Hong Kong 999077 P.R. China; 6 Ultrafast Electron Microscopy Laboratory, School of Physics, Nankai University, Tianjin, 300071 P.R. China; 7 The MOE Key Laboratory of Weak-Light Nonlinear Photonics, School of Physics, Nankai University, Tianjin, 300071 P.R. China

**Keywords:** Materials for optics, Nanoscale materials

## Abstract

Exciton transport in two-dimensional Ruddlesden−Popper perovskite plays a pivotal role for their optoelectronic performance. However, a clear photophysical picture of exciton transport is still lacking due to strong confinement effects and intricate exciton-phonon interactions in an organic-inorganic hybrid lattice. Herein, we present a systematical study on exciton transport in (BA)_2_(MA)_*n*−1_Pb_*n*_I_3*n*+1_ Ruddlesden−Popper perovskites using time-resolved photoluminescence microscopy. We reveal that the free exciton mobilities in exfoliated thin flakes can be improved from around 8 cm^2^ V^−1^ s^−1^ to 280 cm^2^V^−1^s^−1^ by anchoring the soft butyl ammonium cation with a polymethyl methacrylate network at the surface. The mobility of the latter is close to the theoretical limit of Mott-Ioffe-Regel criterion. Combining optical measurements and theoretical studies, it is unveiled that the polymethyl methacrylate network significantly improve the lattice rigidity resulting in the decrease of deformation potential scattering and lattice fluctuation at the surface few layers. Our work elucidates the origin of high exciton mobility in Ruddlesden−Popper perovskites and opens up avenues to regulate exciton transport in two-dimensional materials.

## Abstract (web summary)

Exciton transport in 2D Ruddlesden−Popper perovskite plays a key role for their optoelectronic performance. Here, authors significantly enhance free exciton mobilities in exfoliated thin flakes by anchoring butyl ammonium cation with polymethyl methacrylate, which also improves lattice rigidity.

## Introduction

Two-dimensional (2D) Ruddlesden−Popper perovskites (RPPs) are recently emerging solution-processed perovskite materials that show high promise for photovoltaic and light-emitting applications^1–5^. These materials have a chemical formula (LA)_2_(A)_*n*−1_Pb_*n*_X_3*n*+1_, where A is a small organic monovalent cation, X is a halide, and LA is a large organic spacer cation that separates *n* layers of corner-sharing PbX_6_ octahedra, rendering them natural quantum well (QW) structures. The strong quantum confinement, together with the dielectric confinement set by the spacer layer in these QWs gives rise to strongly bound exciton with large oscillator strengths and binding energies on the order of several hundred meVs^6,7^, making them a versatile platform to understand the physics of 2D excitons.

The 2D excitons, with wavefunctions being delocalized across many unit cells but also exhibiting high binding energies^8^, are distinct from bulk excitons and their transport is intimately tied to the performance of light harvesting and emitting devices based on their host materials. 2D RPPs possess a soft organic-inorganic hybrid lattice with peculiar properties such as strong lattice fluctuation^9^, surface relaxation^10^, dynamic polaron^11^, hot phonon bottleneck^10^, hybrid phonon modes^12^, etc. The presence of these properties adds complexity to the exciton–phonon interactions in 2D RPPs, potentially having a significant impact on their exciton transport properties^13–15^. Other factors such as local energy landscape^16,17^, exciton-exciton annihilation^18^, interlayer coupling^19^ and defects^20^ can also influence exciton transport behavior in RPPs. Therefore, despite much progress, a clear correlation between exciton transport and lattice properties, particularly regarding the exciton-lattice interaction, is still elusive. Moreover, tailoring exciton transport properties by regulating exciton–phonon interactions is urgently needed for 2D perovskite-based optoelectronic applications.

Herein, we directly visualize exciton propagation in a series of exfoliated (BA)_2_(MA)_*n*−1_Pb_*n*_I_3*n*+1_ RPP thin flakes, where BA is butyl ammonium (CH_3_(CH_2_)_3_NH_3_) and MA is methyl ammonium (CH_3_NH_3_), using time-resolved photoluminescence microscopy (TRPLM) with the time-resolution of a few ps and spatial resolution of ~0.22 μm (Supplementary Fig. S1). We observe that the bare exfoliated (BA)_2_PbI_4_ flakes have a modest free exciton mobility of ~7.6 cm^2^ V^−1^ s^−1^, which can be drastically improved to ~280 cm^2^ V^−1^ s^−1^ by capping the surface of thin flakes with a PMMA layer. The exciton mobility is higher by 1–2 orders of magnitude compared to earlier findings and it approaches the Mott-Ioffe-Regel (MIR) limit which defines the transition between the hopping regime and band-like regime^8,21,22^. Temperature-dependent PL measurements reveal that the PMMA may anchor the butyl ammonium molecules at the surface, leading to a more rigid lattice with reduced disorder, which is corroborated by molecular dynamics (MD) simulations. As a result, the exciton–phonon scattering deformation potential decreases from 4.0 × 10^8 ^eV/cm to 9.9 × 10^7 ^eV/cm, which significantly improve the exciton transport. These insights cast light on the strong correlation between exciton transport, lattice rigidity and exciton-lattice interactions, and provides a clear design strategy to customize exciton transport properties in RPPs for optoelectronic applications.

## Results

**Fig. 1:**
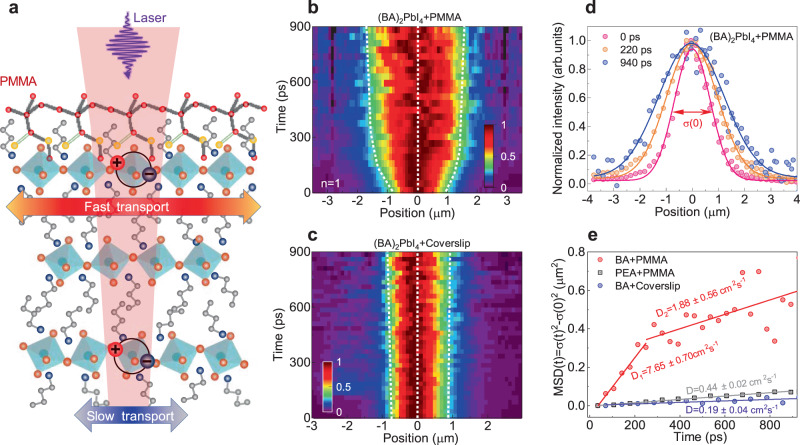
Exciton transport in exfoliated (BA)_2_PbI_4_ RPP flakes encapsulated by coverslip and PMMA. **a** Schematic of TRPLM and exciton transport in RPP flakes with PMMA. Normalized TRPLM image of an (BA)_2_PbI_4_ RPP flake encapsulated by **b** coverslip (w/o PMMA) and **c** PMMA (with PMMA). The *x* axis and *y* axis correspond to the spatial and temporal distributions, respectively. For a better visualization, the TRPL intensity has been normalized to its maximum at each time slice. **d** Spatial distribution of PL at selected time delays for (BA)_2_PbI_4_ RPP flake with PMMA. **e** Diffusion coefficients are present by temporal evolution of the mean-square-displacement MSD (*t*)= σ^2^(*t*)–σ^2^(0) of the exciton at 40 pJ/cm^2^. The (BA)_2_PbI_4_ with coverslip and (PEA)_2_PbI_4_ with PMMA shows a single-step diffusion, which is consistent with previous reports. A two-step diffusion was found for (BA)_2_PbI_4_ with PMMA.

Exciton transport imaging. Exciton transport in (BA)_2_(MA)_*n*−1_Pb_*n*_I_3*n*+1_ RPP flakes was studied using TRPLM technique schematically illustrated in Fig. 1a. In brief, a Gaussian beam was used to excite the sample perpendicularly (in *z*-direction). After photo-excitation, the generated excitons will diffuse out of the excitation volume due to the density gradient^23^, and therefore the emitting area in the *x–y* plane will expand as exciton diffuses, whose dynamics are recorded by a streak camera. The RPP flakes were exfoliated from chemically synthesized large single crystals using a scotch tape and then transferred to a Si/SiO_2_ substrate, which was further encapsulated by a thick layer of PMMA with a thickness of ~300 nm (Supplementary Fig. S2). Control flakes were also prepared by encapsulating the flakes with coverslips in the glovebox. Typical thicknesses of the flakes used in our experiments are in the range of a few hundred nm (Supplementary Fig. S3). Their absorption, PL spectra and XRD pattern are shown in Supplementary Fig. S3, S4.

Representative normalized TRPLM images for (BA)_2_PbI_4_ flake encapsulated by coverslip and PMMA are displayed in Fig. 1b, c. Clearly, the exciton distribution in (BA)_2_PbI_4_ with coverslip shows minor broadening within its lifetime. While a Voigt function may be more accurate^24^, a Gaussian function: $N(r,\, t)={N}_{0}\exp (-({r-{r}_{0}})^{2}/2{\sigma }^{2}(t))$, where ${\sigma }^{2}(t)$ is the time-dependent variance, is more widely used and applied here to extract the exciton distribution. The diffusion coefficient was extracted using the formula:$2Dt=\varDelta {\sigma }^{2}={\sigma }^{2}(t)-{\sigma }^{2}(0)$, where $\varDelta {\sigma }^{2}$ is known as the mean-square displacement. The bare (BA)_2_PbI_4_ flake with coverslip shows a modest diffusion coefficient *D* = 0.19 ± 0.04 cm^2^ s^−1^, corresponding to an exciton mobility of ~7.6 cm^2^ V^−1^ s^−1^. This result is in agreement with recent reports (Table 1). However, for (BA)PbI_4_ with PMMA, the exciton distribution experiences a drastic broadening at the initial 250 ps, followed by a slow one at a later time (Fig. 1d). The fast broadening corresponds to a diffusion coefficient *D*_1_ = 7.65 ± 0.70 cm^2^ s^−1^, while the subsequent slow one correlates to a diffusion coefficient *D*_2_ = 1.88 ± 0.56 cm^2^ s^−1^ (Fig. 1e). As a comparison, we also measured the diffusion coefficient of (PEA)_2_PbI_4_ with PMMA encapsulation, *D* = 0.44 ± 0.02 cm^2^ s^−1^ (Fig. 1e), which does not show significant improvement compared with previous reports w/o PMMA encapsulation^15^. This may be attributed to the structural rigidity and pronounced steric hindrance induced by PEA, which makes PMMA only adsorb on the surface and ineffective to exciton properties in the perovskite layer^25^.

**Table 1:** A literature survey of exciton diffusion coefficients for 2D perovskite measured using a similar technique

Materials	*D* (cm^2^ s^−1^)	μ (cm^2^ V^−1^ s^−1^)	Methods	References	Sample
(PEA)_2_PbI_4_	0.23	13.2	TRPL microscopy	*Materials Horizons* 8, 639, (2021)	Bulk single crystal
(PEA)_2_PbI_4_	1	40	TRPL microscopy	*Nano Lett*. 20, 6674, (2020)	*h*BN/perovskite/*h*BN
(PEA)_2_PbI_4_ (PEA)_2_PbBr_4_	0.222	8.88	TRPL microscopy	*ACS Energy Lett*. 7, 358, (2020)	Perovskite/coverslip/oil immersion objective
(PEA)_2_PbI_4_ (BA)_2_PbI_4_	0.192 0.013	7.68 0.52	TRPL microscopy	*Nat Commun*. 11, 2035, (2020)	Perovskite/glass slide/oil immersion objective
(BA)_2_(MA)_*n*−1_Pb_*n*_I_3*n*+1_ (*n* = 1–5)	0.06–0.34	2.4–13.6	TA microscopy	*Nat Commun*. 11, 664 (2020)	SiO_2_/perovskite/coverslip
(BA)_2_(MA)_*n*−1_Pb_*n*_I_3*n*+1_ (*n* = 1−3) (PEA)_2_(MA)_*n*−1_Pb_*n*_I_3*n*+1_ (*n* = 1−3) (mF-PEA)_2_PbBr_4_	0.058–0.121 0.157–0.648 1.91	0.116–0.484 6.28–25.92 76.4	TPLM	Adv. Mater. 32,46, 2004080 (2020)	coverslip/perovskite/coverslip
(mF-PEA)_2_PbBr_4_	0.35	14.1	TPLM	ACS Energy Lett. 7, 3, 984 (2022)	Bulk single crystal
(BA)_2_(MA)_*n*−1_Pb_*n*_I_3*n*+1_ (*n* = 1–3)	7.1–9.4	280–380	TRPL microscopy	This work	SiO_2_/Perovskite/PMMA

**Fig. 2:**
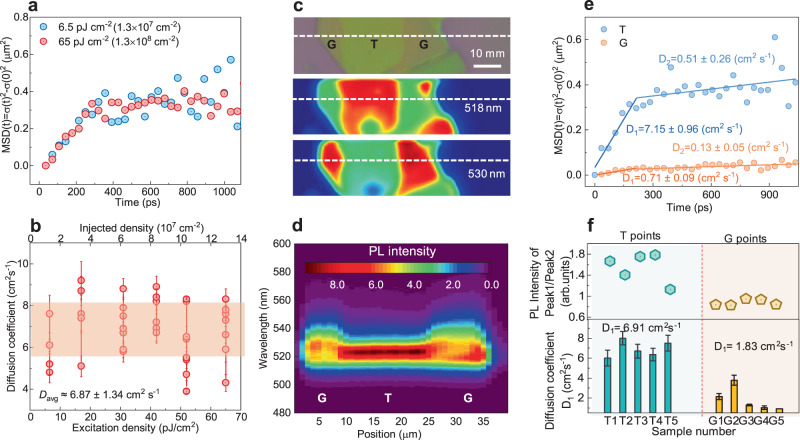
Origin of the fast and slow exciton diffusions for (BA)_2_PbI_4_ flakes with PMMA. **a** MSDs versus time for the lowest and highest excitation density used in our measurements for (BA)_2_PbI_4_ covered with PMMA. **b** Extracted *D*_1_ versus excitation density. The shaded area displays the range of D $\approx$ 6.87 $\pm$ 1.34 cm^2^ s^−1^. The error bar is obtained by linearly fitting the diffusion coefficient using the least squares method. **c** Optical image (up), PL mapping at 518 nm (middle) and at 530 nm (down) of a (BA)_2_PbI_4_ flake where G and T regions are observed. **d** The PL spectra scan across the dash lines in Fig. 3a. **e** Representative MSDs of exciton diffusion at T and G regions. **f** Correlation between the weight of two peaks in PL and diffusion coefficients for T and G regions.

We note that in an early work by some of us, the excitation density reaches 10^11^−10^12^ cm^−2^, which results in a notable contribution from high-order recombination and a slowdown of exciton distribution expansion at a longer time^18^ However, the excitation density in our work is in the range of 10^7^−10^8^ cm^−2^, which are 4 orders lower than those of the early work. Given a bimolecular recombination coefficient *k*_2_ of around 10^−2^ cm^2^ s^−1 ^^18^, such a low exciton density yields an effective lifetime of 1–10 μs, a value that is a few orders lower than that of monomolecular recombination lifetime of ~100 ps. This is consistent with the excitation density independent exciton diffusion coefficients (Fig. 2a, b) and lifetimes (Supplementary Fig. S5) in our measurements. Therefore, the two-step-like diffusion is not caused by high-order recombinations^14,18^. Furthermore, we also ruled out the influence of hot excitons. In transient absorption (TA) kinetics, a fast decay with a time constant of 0.5 ± 0.2 ps is attributed to the relaxation of hot excitons^26^ (Supplementary Fig. S5). The hot exciton diffusion should happen within the temporal resolution of TRPLM setup, and therefore, it is not likely to contribute to the observed exciton transport dynamics.

We observed that for thick exfoliated flakes (>300 nm), the tail of PL spectrum can become a peak, which results in two well-separated peaks: a high energy (HE) peak at around 520 nm and a low energy (LE) peak at around 526–528 nm (Supplementary Fig. S3). The LE peak is consistent with the emission upon two-photon excitation, which can penetrate deep into the bulk (Supplementary Fig. S6)^27^. Therefore, we can attribute the LE peak to bulk emission, which is redshifted, most probably due to significant reabsorption for a thick sample. Figure 2c, d shows a representative microscopic image of an exfoliated flake and its PL mapping at two wavelengths (518 nm and 530 nm) close to the HE and LE peaks, respectively. We denote the regions with higher HE peaks as T regions and those with a higher LE peak as G regions. Here the T region and G region have an average thickness of around 180 nm and 400 nm, respectively. Figure 2e, f shows that the exciton diffusion at T regions is superior to that at G regions. For G regions, the coefficient of the fast diffusion almost approaches that of the slow one leading to a similar trend with that w/o PMMA (Fig. 2e, f and Supplementary Fig. S7). From these results, we can rationalize that the effect of PMMA on thick flakes are minor, and therefore the fast diffusion *D*_1_ corresponds to the excitons generated close the surface whose transport properties are strongly affected by the presence of PMMA. The slow diffusion *D*_2_, on the other hand, correlates to those excitons generated in the bulk that are less affected by PMMA. The bulk excitons have a longer lifetime (>ns) than those surface excitons, implying that PMMA does not simply act as a defect passivation layer to reduce the non-radiative recombination. Instead, these excitons may easily be localized by strong exciton–phonon coupling forming long-lived species with exciton-polaron (self-trapped exciton) characteristics^28^, which is, however, much alleviated at the surface by PMMA. We should stress that the interplane diffusion takes place simultaneously with the measured intraplane diffusion through TRPLM^19^. This leads to the diffusion of surface excitons into the bulk, dynamically transforming them into bulk excitons. Theoretical simulations demonstrate that this type of diffusion brings about a smooth transition from fast to slow diffusion regimes like what we observed in this work, instead of an abnormal contraction observed for isolated species in the presence of distributed trap states (Supplementary Fig. S8)^29^.

**Fig. 3:**
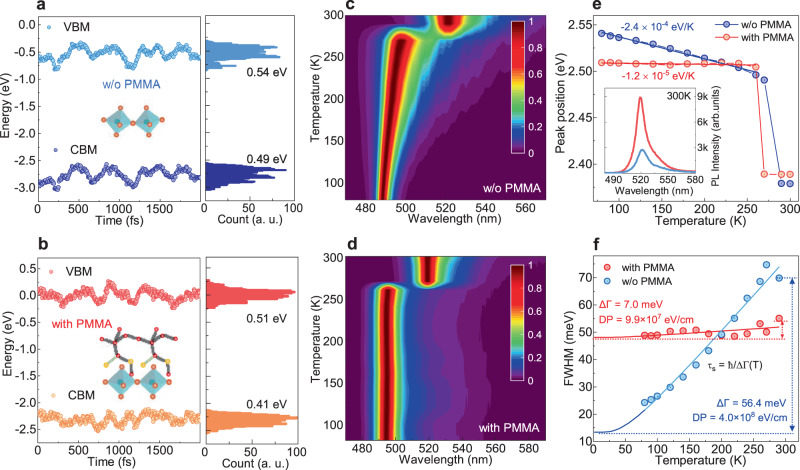
Temperature-dependent PL and deformation potential. Band energy fluctuations for (BA)_2_PbI_4_ **a** w/o and **b** with PMMA. The statistical results are shown on the right side of the figures. Temperature-dependent PL for (BA)_2_PbI_4_ flakes **c** w/o and **d** with PMMA. The PL intensity has been normalized for clarity. **e** Temperature-dependent PL peak position of (BA)_2_PbI_4_ flakes with and w/o PMMA. The inset is the corresponding PL spectra at 300 K. **f** Temperature-dependent PL FWHM of (BA)_2_PbI_4_ flakes with and w/o PMMA.

We performed molecular dynamics simulations to unveil the mechanisms of exciton transport improvement for (BA)PbI_4_ flakes with PMMA. Figure 3a, b shows representative band gap fluctuations over time during the ab initio MD simulations for (BA)_2_PbI_4_ w/o and with PMMA layer. We observe that the CBM and VBM of (BA)_2_PbI_4_ strongly fluctuate over a range of 0.54 eV and 0.49 eV, respectively, which agrees well with recent reports on its large band gap fluctuation^28^. For (BA)_2_PbI_4_ with PMMA, the band energy fluctuations decrease to 0.51 eV and 0.41 eV for the CBM and VBM, respectively. The large PMMA polymer can well penetrate into the aliphatic BA molecule layer, serving as an anchor to stabilize it and decrease its structural disorder. On the other hand, (PEA)_2_PbI_4_ possesses a more rigid lattice due to the formation of an extensive network of pi-hydrogen bonds and a more space-filling nature of the aromatic ring, which is less sensitive to the presence of PMMA^15^. We also note that the band energy fluctuation of the VBM is more affected by PMMA. This can be rationalized considering the orbital characteristics at the band edges. The VBM of 2D lead iodide perovskites is primarily composed of 5p orbitals from I and 6 s orbitals from Pb, while the CBM of 6p orbitals from Pb^30^. The lattice fluctuations mainly originate from the change of Pb-I bond, e.g., octahedra tilting, and therefore are strongly reduced with PMMA.

Lattice rigidity strongly correlates with exciton–phonon coupling, which impacts exciton mobility and emission properties^15^. To pinpoint the mechanisms of exciton–phonon interaction in exfoliated (BA)_2_PbI_4_ flakes, we further conducted PL studies at varying temperatures. As shown in Fig. 3c, d and Supplementary Fig. S9, the temperature evolutions of PL spectra for the exfoliated (BA)_2_PbI_4_ flake w/o and with PMMA are strikingly different. Firstly, we observed significant PL intensity enhancement in the sample with PMMA (Fig. 3e inset), which is possibly related to the greater lattice rigidity caused by PMMA and suppressed exciton–phonon coupling^13,15,31^. Secondly, the PL position for the (BA)_2_PbI_4_ flake w/o PMMA displays an obvious redshift with the increase in temperature. The trend is opposite to that of 3D perovskites. Lattice dilation and electron-phonon coupling are two dominant mechanisms for the temperature dependence of the band gap^32^. In typical 3D perovskites, the effect of lattice dilation is more important, which leads to the increase of band gap with temperature. For (BA)_2_PbI_4_ flake, the electron/exciton–phonon coupling dominates, resulting in a reverse effect. The coefficient of the temperature-dependence of the band gap (d*E*_g_/d*T*) is fitted to be −2.4 ± 0.1 × 10^−4 ^eV/K below 270 K. An abrupt change in PL position occurs at around 270 °C, which is attributed to the phase transition driven by the ordering of the alkyl-ammonium^33^. However, the phase transition temperature for the exfoliated flakes is higher than that of the bulk one, which reveals that it is more difficult for the surface part to undergo phase transition^34^. On the other hand, the PL peak position is almost invariant for the flake with PMMA before the phase transition temperature, with the temperature-dependent coefficient (d*E*_g_/d*T*) being only −1.2 ± 0.6 × 10^−5 ^eV/K below the phase transition temperature. These results highlight that PMMA may efficiently stabilize the soft surface and reduce the exciton–phonon coupling strength. Lastly, as shown in Fig. 3f, the PL bandwidth for flake with PMMA displays a much reduced dependence on temperature than that of flake w/o PMMA. The thermal broadening of PL bandwidth is mainly attributed to the increased phonon scattering with increasing temperature. Two main mechanisms can account for the interaction between exciton and lattice vibrations in lead halide perovskites: Fröhlich polar interaction and deformation potential scattering^35,36^. In quasi-2D perovskites, the local electric field by electron and hole in a strongly confined 2D exciton may cancel each other, leading to a weak Fröhlich polar interaction^37^. Therefore, deformation potential scattering via acoustic phonon or homopolar optical phonon is usually considered the main exciton–phonon interaction mechanism in quasi-2D perovskites^38^. The PL bandwidth (full-width at half-maximum) is fitted using the expression^38^:

1 $$\varGamma (T)={\varGamma }_{0}+\frac{\hslash M{\omega }_{{{{{{\rm{o}}}}}}}}{\rho L}{\left(\frac{{D}_{{{{{{\rm{cv}}}}}}}}{\hslash {\omega }_{{{{{{\rm{o}}}}}}}}\right)}^{2}(2-{e}^{-\hslash {\omega }_{{{{{{\rm{o}}}}}}}/{k}_{{{{{{\rm{B}}}}}}}T})/({e}^{\hslash {\omega }_{{{{{{\rm{o}}}}}}}/{k}_{{{{{{\rm{B}}}}}}}T}-1)$$

where *Γ*_0_ is the inhomogeneous broadening at zero temperature, *M* is the sum of electron and hole effective masses; *ρ* is the mass density, *L* is the thickness of the QW, *ω*_o_ is the angular frequency of the homopolar optical phonon, and *D*_cv_ is the deformation potential. For (BA)_2_PbI_4_, *M* = 0.5 *m*_0_, *L* = 0.6 nm, *ρ* = 2.8 g/cm^3 ^^31^. We also used the homopolar phonon frequency of 94 cm^−1^ for the fitting, which is observed in Raman spectrum (Supplementary Fig. S10) and assigned to the homopolar phonon modes of the out-of-plane I–Pb–I stretch^38^. We estimate that the deformation potential *D*_cv_ decreases from 4.0 × 10^8^ eV/cm to 9.9 × 10^7^ eV/cm by PMMA. Therefore, the greater lattice rigidity and the smaller deformation potential caused by PMMA rigid network is likely the reason for the increase in exciton mobility.

**Fig. 4:**
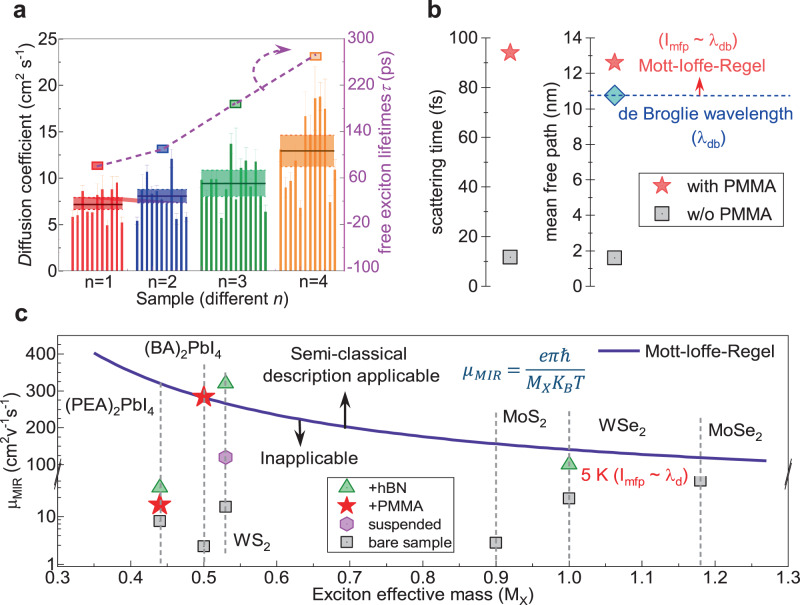
Exciton diffusion approaching MIR limit. **a** Histogram of the fast exciton diffusion coefficients *D*_1_ for (BA)_2_(MA)_*n*−1_Pb_*n*_I_3*n*+1_ (*n* = 1−4) RPP flakes at 40 pJ/cm^2^. The free exciton lifetimes for these flakes are also plotted. The shaded area represents the average range of diffusion coefficients D corresponding to different *n*. The error bar is obtained by linearly fitting the diffusion coefficient using the least squares method. **b** Calculated mean free path for (BA)_2_PbI_4_ RPP flakes with and w/o PMMA. **c** Comparison of experimentally measured mobilities and MIR mobilities for different 2D materials.

To generalize our findings, we also measured the exciton transport for different layer number *n*. Similar to the *n* = 1 with coverslip, the diffusion coefficients are 0.33 cm^2^ s^−1^ and 0.40 cm^2^ s^−1^ for *n* = 2 and 3 samples, respectively, in consistency with recent reports (Table 1 and Supplementary Fig. S11). The diffusion coefficients increase notably with PMMA, with the averaged fast diffusion coefficients *D*_1_ reaching 8.0 cm^2^ s^−1^, 9.4 cm^2^ s^−1^ and 12.9 cm^2^ s^−1^ for *n* = 2, 3 and 4 samples, respectively (Fig. 4a). The trend of exciton mobility correlates with the lifetime increase: 110 ps, 186 ps and 273 ps for *n* = 2, 3, 4, respectively (Fig. 4a and Supplementary Fig. S12). This is due to a less quantum confinement effect and a larger fraction of free charge carriers generated in larger *n* R-P perovskites.

## Discussion

Exciton diffusion can be generally categorized into two mechanisms: semiclassical band-like transport for Wannier-Mott excitons in inorganic semiconductors or hopping between localization sites for Frenkel excitons in molecular crystals^8^. Exciton transport at the R-P perovskite organic-inorganic hybrid lattice with strong confinements may be the intermediate case. Indeed, using the deformation potentials extracted from PL bandwidth measurements, we obtained scattering times ${\tau }_{{{{{{\rm{s}}}}}}}=\hslash /\varDelta \varGamma$ of 11.7 fs and 94.0 fs for (BA)_2_PbI_4_ w/o and with PMMA, respectively. The exciton mean free path is given by: ${l}_{{{{{{\rm{mfp}}}}}}}=\sqrt{2D{\tau }_{{{{{{\rm{s}}}}}}}}$, and are around 1.6 nm and 12.6 nm for (BA)_2_PbI_4_ w/o and with PMMA, respectively (Fig. 4b). The thermal de Broglie wavelength of exciton is expressed as:${\lambda }_{{{{{{\rm{db}}}}}}}=\sqrt{2}\pi \hslash /\sqrt{{M}_{{{{{{\rm{X}}}}}}}{k}_{{{{{{\rm{B}}}}}}}T}$, where *M*_X_ = *m*_e_ + *m*_h_ is the exciton mass. For (BA)_2_PbI_4_, ${\lambda }_{{{{{{\rm{db}}}}}}}$ ~10.8 nm is much larger than the mean free path of excitons w/o PMMA. This implies that the conventional semiclassical description of exciton transport in 2D bare (BA)_2_PbI_4_ flakes is inadequate, as it necessitates an inelastic scattering rate that is much less than the thermal energy of excitons. This criterion is also referred to as the MIR criterion: ${\lambda }_{{{{{{\rm{db}}}}}}} \sim {l}_{{{{{{\rm{mfp}}}}}}}$. For the limiting case, i.e., ${\lambda }_{{{{{{\rm{db}}}}}}}={l}_{{{{{{\rm{mfp}}}}}}}$, one can obtain a mobility limit that is only related to the exciton mass and temperature^14^:

2 $${\mu }_{{{{{{\rm{MIR}}}}}}}=\frac{e\pi \hslash }{{M}_{{{{{{\rm{X}}}}}}}{k}_{{{{{{\rm{B}}}}}}}T}$$

The MIR limit can be used to evaluate whether exciton transport is more likely by hopping or free propagation. In general, for most 2D materials without any surface modification, the exciton transport falls below the MIR limit (Fig. 4c)^8,13–15,18,39–44^. For (BA)_2_PbI_4_ w/o PMMA, scattering seems to occur on a length scale of only two or three unit cells and destroys the coherence of exciton^45^. Additional effects including strong localization, hopping and polarons need to be included beyond the semiclassical approach^8,22,46^. For (BA)_2_PbI_4_ with PMMA, the surface exciton has${\lambda }_{{{{{{\rm{db}}}}}}} \sim {l}_{{{{{{\rm{mfp}}}}}}}$, which indicates that exciton propagation approaches the MIR limit (*μ*_MIR_ = 289 cm^2^ V^−1^ s s^−1^ for *M*_X_ = 0.5 *m*_0_) and can be roughly described by the band-like transport (Fig. 4b). Indeed, using the experimentally determined scattering time *τ*_s_ = 94.0 fs above, the semiclassical approach yields an exciton mobility $\mu=e{\tau }_{{{{{{\rm{s}}}}}}}/{M}_{{{{{{\rm{X}}}}}}}$ of ~340 cm^2^ V^−1^ s^−1^, close to the measured exciton mobility. This highlights the importance of surface reconstruction to tailor the lattice rigidity to regulate exciton propagation from the hopping regime to the band-like transport regime. This surface lattice reconstruction by organic molecules is strongly affected by the bonding strength, intercalation distance, organic backbone rigidity, etc. For example, introducing chelation, π-conjugation, polymerization have already been shown to exert profound effects on the carrier transport of perovskite materials^25,47,48^. Future research should focus on how to optimize these parameters in order to further improve the exciton transport in these materials.

In summary, we have directly monitored ultrafast exciton propagation process in (BA)_2_(MA)_*n*-1_Pb_*n*_I_3*n*+1_ R-P perovskites and observed that the exciton transport can be significantly improved to the theoretical limit of MIR criterion with PMMA encapsulation. We reveal the enhanced exciton mobility is attributed to the anchoring of surface BA molecules by PMMA network, which significantly improve the lattice rigidity and reduces the disorder. As a result, the deformation potential scattering rate reduces by 8 times at room temperature, leading to the transition of exciton propagation from the hopping regime to the band-like transport regime. These findings provide insights into the mechanisms of exciton transport in 2D perovskites with a soft lattice and shed light on how to tune exciton transport properties through lattice engineering.

## Methods

Growth of (BA)_2_(MA)_*n*−1_Pb_*n*_I_3*n*+1_ (*n* = 1−4) single crystal: First, *n*-BA (462 μL, 5 mmol, *n* = 1; 347 μL, 3.5 mmol, *n* = 2; 163.5 μL, 1.665 mmol, *n* = 3; 124 μL, 1.25 mmol, *n* = 4) is reacted with excess HI acid (2.5 mL, 19 mmol) in an ice bath to obtain a BAI precursor solution. In another beaker in oil bath at 100 °C, PbO powder (1116 mg, 5 mmol) was dissolved in a mixture of H_3_PO_2_ (0.85 mL, 7.75 mmol) and HI acid (5.0 mL, 38 mmol) by continuous magnetic agitation until a yellow PbI_2_ precursor solution was formed. Addition of MACl (0 mg, 0 mmol, *n* = 1; 338 mg, 5 mmol, *n* = 2; 450 mg, 6.67 mmol, *n* = 3; 507 mg, 7.5 mmol, *n* = 4) to PbI_2_ precursor solution, which promotes crystallization but does not participate in the reaction. Subsequent addition of the BAI precursor, the resulting precipitation was dissolved in the 100 °C-oil bath by continuous magnetic agitation until a clear and bright yellow precursor solution was formed. Finally, the agitation was stopped, the solution was cooled to room temperature, and the single crystal was separated by suction filtration^49,50^.

Sample preparation of (BA)_2_(MA)_*n*-1_Pb_*n*_I_3*n*+1_ (*n* = 1 − 4) with PMMA: We dissolved PMMA particles (Polymethyl methacrylate, P821346-100g, MACKLIN Inc.) in toluene to prepare 100 mg/ml solution which was then stored in the refrigerator. For deposition, A volume of 40 mL PMMA solution is spin-coated onto a 1 × 1 cm substrate with (BA)_2_(MA)_*n*−1_PbnI_3*n*+1_ (*n* = 1–4) at a speed of 500 rpm for 5 s followed by 4000 rpm for 60 s. The sample is then dried in the glovebox for 12 hrs before measurement.

Steady-state absorption spectroscopy: The continuous white light from a halogen lamp source (SLS201L, broadband 360−2600 nm) focused with a ×60 objective lens onto the sample. The spectra were coupled into a Princeton Instrument SP2500i spectrometer with a liquid nitrogen-cooled charge-coupled device detector (CCD) by an optical fiber.

Steady-state Photoluminescence and Raman measurement: The PL spectra were excited by a 405 nm CW laser focusing on the (BA)_2_(MA)_*n*−1_PbnI_3*n*+1_ RPP flake with a ×100 objective lens (Olympus, ×100, NA = 0.9). The Raman spectra were excited by a 633 nm CW laser. The size of the laser beam is ~2 μm, which was focused on the (BA)_2_(MA)_*n*−1_PbnI_3*n*+1_ RPP flake using the same objective lens. Steady-state Photoluminescence and Low-frequency Raman spectra were collected by Horiba-JY T64000.

Transient absorption spectroscopy: The femto-second (800 nm, 100 fs, 1 kHz, Astrella, Coherent) TA measurements were obtained by the HELIOS commercial fs-TA system (Ultrafast Systems). Different wavelengths of pump laser were obtained by an optical parametric amplifier (Coherent, OperA Solo). The probe beam of continuum white light is about from 450 to 750 nm (1.65 eV–2.75 eV).

TRPLM and TRPL Measurements: The exciton diffusion of TRPL microscopy and power-dependent TRPL were collected by a steak camera (Optronis SC-10-OptoScope). The excitation 400 nm pulsed laser is obtained by frequency doubling by 800 nm (100 fs, 1 kHz, Astrella, Coherent) with a BBO crystal. Exciton diffusion measurements (TRPLM) were measured by PL of the exciton population imaging on CMOS camera of steak camera. We used a lens group to eliminate distortion and obtained uniformly spaced ruler imaging. As shown in Figure S1, the spatial calibration of the microscope image was performed using a commercial calibration standard ruler. The microscope objective used was a ×100 objective (*N*_A_ = 0.9), and the spatial resolution of the measurement system is about *λ*/(2*N*_A_) = 400/(2 × 0.9) = 0.22 μm.

Computational Methods: The exchange-correlation energy was approximated using the generalized gradient approximation (GGA) method with Perdew-Burke-Ernzerhof (PBE) functional as implemented in the Quantum ESPRESSO package. The electron-ion interactions were described by ultrasoft pseudopotentials by treating electrons for H (1*s*^1^), C (2*s*^2^, 2*p*^2^), N (2*s*^2^, 2*p*^3^), O (2*s*^2^, 2*p*^4^), I (5*s*^2^, 5*p*^2^), and Pb (5*d*^10^, 6*s*^2^, 6*p*^2^). The single-particle wave functions (charges) were expanded on a plane-wave basis set with a kinetic energy cutoff of 40 Ry (200 Ry) for all slab structures. The van der Waals functional vdW-DF2, which self-consistently accounts for dispersion effects was also used for the structural optimizations. Monkhorst-Pack type *k*-meshes of 4 × 4 × 2 for the bulk (BA)_2_PbI_4_ and 4 × 4 × 1 for (BA)_2_PbI_4_ slabs exposing the (100) surface was used. All the symmetric slabs separated by a vacuum layer (~15 Å) to prevent spurious interslab interactions. All the structure were optimized until the forces on each single atom were smaller than 0.01 eV/Å. Molecular dynamics (MD) simulations were conducted at the GGA/PBE level using the same ultrasoft pseudopotentials. The optimized crystal structures of (BA)_2_PbI_4_ (100) slabs without and with PMMA adsorption were considered as the starting point for the MD simulations. The simulations began with an initial 1000 fs trajectory using a 1 fs time step for nuclear thermalization, followed by a 2000 fs production run of the system. The Andersen thermostat was used to control the temperature of the (BA)_2_PbI_4_ slabs without and with PMMA adsorption at 300 K.

### Supplementary information

Supplementary Information

## Data Availability

All data to evaluate the conclusions are present in the manuscript and the Supplementary Material. Raw data are available from the corresponding authors on request.

## References

[1] Tsai H (2016). High-efficiency two-dimensional Ruddlesden–Popper perovskite solar cells. Nature.

[2] Wang N (2016). Perovskite light-emitting diodes based on solution-processed self-organized multiple quantum wells. Nat. Photonics.

[3] Ren H (2020). Efficient and stable Ruddlesden–Popper perovskite solar cell with tailored interlayer molecular interaction. Nat. Photonics.

[4] Yuan M (2016). Perovskite energy funnels for efficient light-emitting diodes. Nat. Nanotechnol..

[5] Shrestha S (2022). Long carrier diffusion length in two-dimensional lead halide perovskite single crystals. Chem.

[6] Blancon JC (2018). Scaling law for excitons in 2D perovskite quantum wells. Nat. Commun..

[7] Mauck CM (2019). Excitons in 2D organic–inorganic halide perovskites. Trends Chem..

[8] Wagner K (2021). Nonclassical exciton diffusion in monolayer WSe_2_. Phys. Rev. Lett..

[9] Quan LN (2021). Vibrational relaxation dynamics in layered perovskite quantum wells. Proc. Natl. Acad. Sci..

[10] Jia X (2018). Observation of enhanced hot phonon bottleneck effect in 2D perovskites. Appl. Phys. Lett..

[11] Tao W (2022). Dynamic exciton polaron in two-dimensional lead halide perovskites and implications for optoelectronic applications. Acc. Chem. Res..

[12] Yin J (2019). Tuning hot carrier cooling dynamics by dielectric confinement in two-dimensional hybrid perovskite crystals. ACS Nano.

[13] Xiao X (2020). Ultrafast exciton transport with a long diffusion length in layered perovskites with organic cation functionalization. Adv. Mater..

[14] Ziegler JD (2020). Fast and anomalous exciton diffusion in two-dimensional hybrid perovskites. Nano Lett..

[15] Seitz M (2020). Exciton diffusion in two-dimensional metal-halide perovskites. Nat. Commun..

[16] Seitz M (2022). Halide mixing inhibits exciton transport in two-dimensional perovskites despite phase purity. ACS Energy Lett..

[17] Baldwin A (2021). Local energy landscape drives long-range exciton diffusion in two-dimensional halide perovskite Semiconductors. J. Phys. Chem. Lett..

[18] Deng S (2020). Long-range exciton transport and slow annihilation in two-dimensional hybrid perovskites. Nat. Commun..

[19] Magdaleno AJ (2021). Efficient interlayer exciton transport in two-dimensional metal-halide perovskites. Mater. Horiz..

[20] Zhao C (2020). Trap-enabled long-distance carrier transport in perovskite quantum wells. J. Am. Chem. Soc..

[21] Giovanni D (2021). Origins of the long-range exciton diffusion in perovskite nanocrystal films: photon recycling vs exciton hopping. Light: Sci. Appl..

[22] Glazov MM (2020). Quantum interference effect on exciton transport in monolayer semiconductors. Phys. Rev. Lett..

[23] Wen X (2022). New insight into carrier transport in 2D layered perovskites. Chem.

[24] Akselrod GM (2014). Subdiffusive exciton transport in quantum dot solids. Nano Lett..

[25] Xue J (2021). Reconfiguring the band-edge states of photovoltaic perovskites by conjugated organic cations. Science.

[26] Wu XX (2015). Excitonic many-body interactions in two-dimensional lead iodide perovskite Quantum wells. J. Phys. Chem. C..

[27] Leng K (2018). Molecularly thin two-dimensional hybrid perovskites with tunable optoelectronic properties due to reversible surface relaxation. Nat. Mater..

[28] He H (2016). Exciton localization in solution-processed organolead trihalide perovskites. Nat. Commun..

[29] Seitz M (2021). Mapping the trap-state landscape in 2D metal-halide perovskites using transient photoluminescence microscopy. Adv. Opt. Mater..

[30] Thouin F (2019). Phonon coherences reveal the polaronic character of excitons in two-dimensional lead halide perovskites. Nat. Mater..

[31] Gong X (2018). Electron–phonon interaction in efficient perovskite blue emitters. Nat. Mater..

[32] Singh S (2016). Effect of thermal and structural disorder on the electronic structure of hybrid perovskite semiconductor CH_3_NH_3_PbI_3_. J. Phys. Chem. Lett..

[33] Ishihara T (1990). Optical properties due to electronic transitions in two-dimensional semiconductors (C_n_H_2n+1_NH_3_)_2_PbI_4_. Phys. Rev. B.

[34] Yaffe O (2015). Excitons in ultrathin organic-inorganic perovskite crystals. Phys. Rev. B.

[35] Herz LM (2017). Charge-carrier mobilities in metal halide perovskites: fundamental mechanisms and limits. ACS Energy Lett..

[36] Ghosh D (2020). Polarons in halide perovskites: a perspective. J. Phys. Chem. Lett..

[37] Feldstein D (2020). Microscopic picture of electron-phonon interaction in two-dimensional halide perovskites. J. Phys. Chem. Lett..

[38] Guo Z (2016). Electron–phonon scattering in atomically thin 2D perovskites. ACS Nano.

[39] Nayak D (2023). Strain modulated electronic and photocatalytic properties of MoS_2_/WS_2_ heterostructure: A DFT study. ACS Appl. Electron. Mater..

[40] Kulig M (2018). Exciton diffusion and halo effects in monolayer semiconductors. Phys. Rev. Lett..

[41] Zipfel J (2020). Exciton diffusion in monolayer semiconductors with suppressed disorder. Phys. Rev. B.

[42] Liu H (2020). Direct visualization of exciton transport in defective few-layer WS_2_ by ultrafast microscopy. Adv. Mater..

[43] Saito K (2021). Femtosecond photoluminescence from monolayer MoS_2_: time-domain study on exciton diffusion. Phys. Rev. B.

[44] Kuhn H (2020). Excitonic transport and intervalley scattering dynamics in large‐size exfoliated MoSe_2_ monolayer investigated by heterodyned transient grating spectroscopy. Laser Photonics Rev..

[45] Ioffe A., et al. Non-crystalline, amorphous, and liquid electronic semiconductors. *Progress in Semiconductors* (Heywood & Company LTD., London, 1960).

[46] Mishchenko AS (2019). Polaron mobility in the “Beyond Quasiparticles” regime. Phys. Rev. Lett..

[47] He J (2020). Surface chelation of cesium halide perovskite by dithiocarbamate for efficient and stable solar cells. Nat. Commun..

[48] Chen W (2021). Polymerized hybrid perovskites with enhanced stability, flexibility, and lattice rigidity. Adv. Mater..

[49] Liang Y (2019). Lasing from mechanically exfoliated 2D homologous Ruddlesden-Popper perovskite engineered by inorganic layer thickness. Adv. Mater..

[50] Stoumpos CC (2016). Ruddlesden–Popper hybrid lead iodide perovskite 2D homologous semiconductors. Chem. Mater..

