# Supplementary material for: Boosting exciton mobility approaching Mott-Ioffe-Regel limit in Ruddlesden−Popper perovskites by anchoring the organic cation
Source: Nat Commun. 2024 Feb 29;15:1893. doi: 10.1038/s41467-024-45740-y (PMC10904778; doi:10.1038/s41467-024-45740-y)
Supplement: Supplementary file 1 — Supplementary Information [file 41467_2024_45740_MOESM1_ESM.pdf]

Supplementary Information for

Boosting Exciton Mobility Approaching Mott-Ioffe-Regel Limit in Ruddlesden−Popper Perovskites by Anchoring the Organic Cation

Yiyang Gong[1,2,3], Shuai Yue[2,3], Yin Liang[4], Wenna Du[2,3], Tieyuan Bian[5], Chuanxiu Jiang[2,3], Xiaotian Bao[2], Shuai Zhang[2,3], Mingzhu Long[1], Guofu Zhou[1], Jun Yin[5], Shibin Deng[6,7], Qing Zhang[4*], Bo Wu[1,2*], and Xinfeng Liu[2,3*]

[1]South China Academy of Advanced Optoelectronics, South China Normal University, Guangzhou 510006, P. R. China

[2]CAS Key Laboratory of Standardization and Measurement for Nanotechnology, National Center for Nanoscience and Technology, Beijing 100190, P.R. China

[3]University of Chinese Academy of Sciences, Beijing 100049, P.R. China

[4]School of Materials Science and Engineering, Peking University, Beijing 100871, P.R. China

[5]Department of Applied Physics, The Hong Kong Polytechnic University, Hung Hom, Kowloon, Hong Kong 999077, P. R. China

[6]Ultrafast Electron Microscopy Laboratory, School of Physics, Nankai University, Tianjin 300071, P.R. China

[7]The MOE Key Laboratory of Weak-Light Nonlinear Photonics, School of Physics, Nankai University, Tianjin 300071, P.R. China

*Email address: liuxf@nanoctr.cn, bowu@m.scnu.edu.cn, Q_zhang@pku.edu.cn

Table of Contents

Figure S1| The spatial calibration of TRPLM.

Figure S2| AFM profiles of the spin-coated PMMA layer.

Figure S3| AFM profiles of the exfoliated *n*=1 RPP flakes.

Figure S4| Absorption and emission spectra of $(BA)_2(MA)_{n-1}Pb_nI_{3n+1}$ RPP flakes.

Figure S5| TRPL and TA dynamics of $(BA)_2PbI_4$ flake upon 400 nm excitation.

Figure S6| TRPL and TA dynamics of $(BA)_2PbI_4$ flake upon 400 nm excitation.

Figure S7| Slow diffusion coefficients D_2 for T and G regions.

Figure S8| Theoretical simulation of the two-step diffusion.

Figure S9| Temperature dependent PL for $(BA)_2PbI_4$ flakes w/o and with PMMA.

Figure S10| Raman spectra of (a) n = 1 RPP flake at 77 K and (b) n = 2 RPP flake at room temperature.

Figure S11| Mean-square-displacement of the exciton population over time of different sample in exfoliated $(BA)_2(MA)_{n-1}PbnI_{3n+1}$ RPP flakes.

Figure S12| Transient absorption dynamics of n = 1-4 RPP exfoliated flakes monitored at the photo-bleaching region.

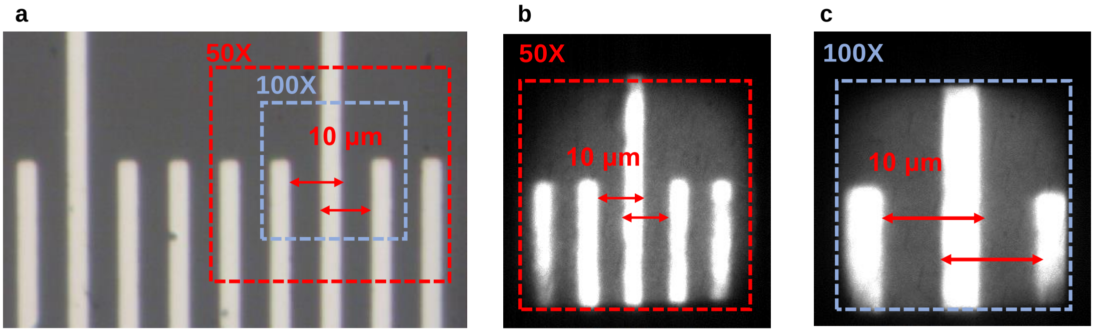

Figure S1| The spatial calibration of TRPLM. (a) Optical imaging of a standard ruler. The scale bar is 10 μm. We imaged the area within the red box in the CCD for calibration of the measurement system. We used a lens group to eliminate distortion and obtained uniformly spaced ruler imaging. Imaging ruler on the CCD of streak camera by **(b)** 50X and **(c)** 100X objective lenses. (10 μm occupy 108 pixel).

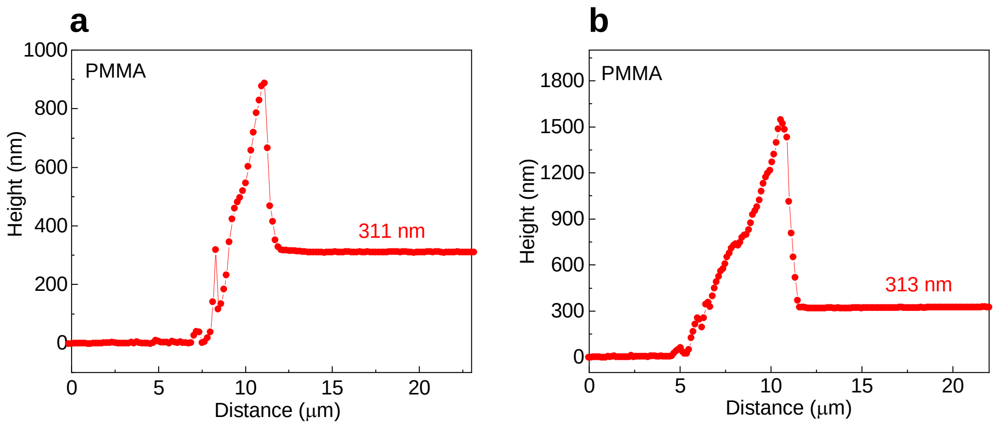

Figure S2| AFM profiles of the spin-coated PMMA layer. The height profiles of a sample scanned from the part w/o PMMA layer to that w/ PMMA layer are shown in (a) and (b) for two representative regions. This indicates the thickness of PMMA layer is around 310 nm. The hump at the boundary is caused by the removal of PMMA for AFM measurement.

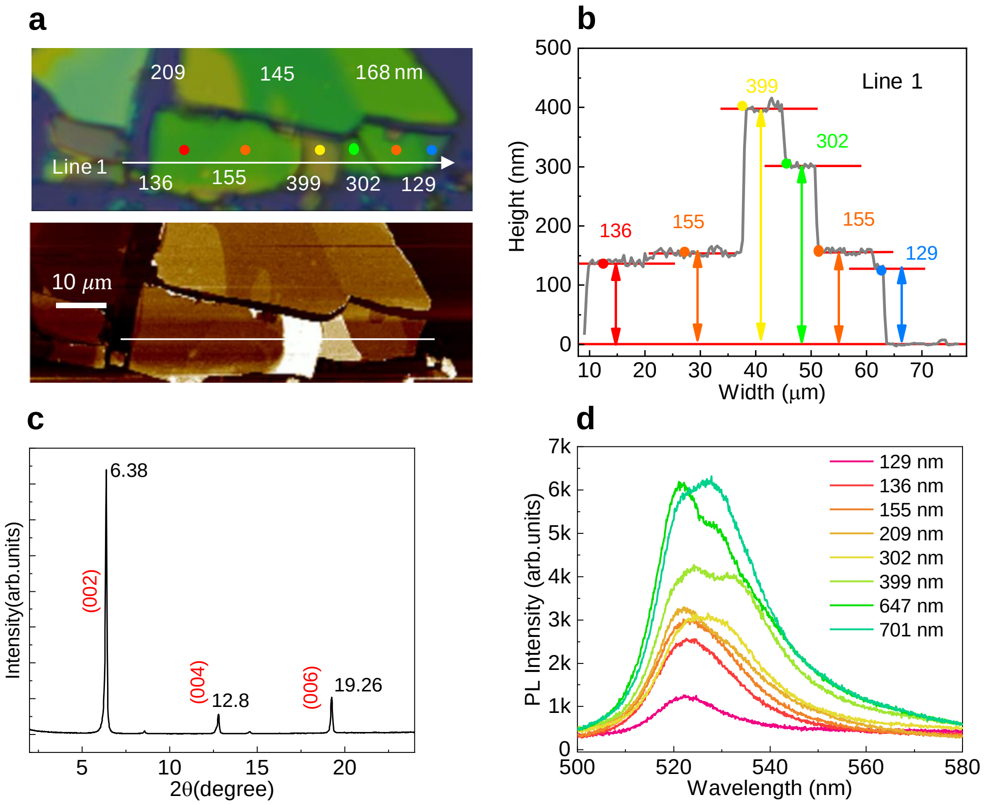

Figure S3| AFM profiles of the exfoliated *n*=1 RPP flakes. (a) Up: microscopic image, bottom: AFM image. (b) AFM profiles scanned along the white line shown in figure a. (c) The XRD pattern of exfoliated $(BA)_2PbI_4$ flake. The (002), (004) and (006) diffraction peaks are located at 6.38°, 12.80° and 19.26°, respectively. (d) Thickness-dependent PL spectra.

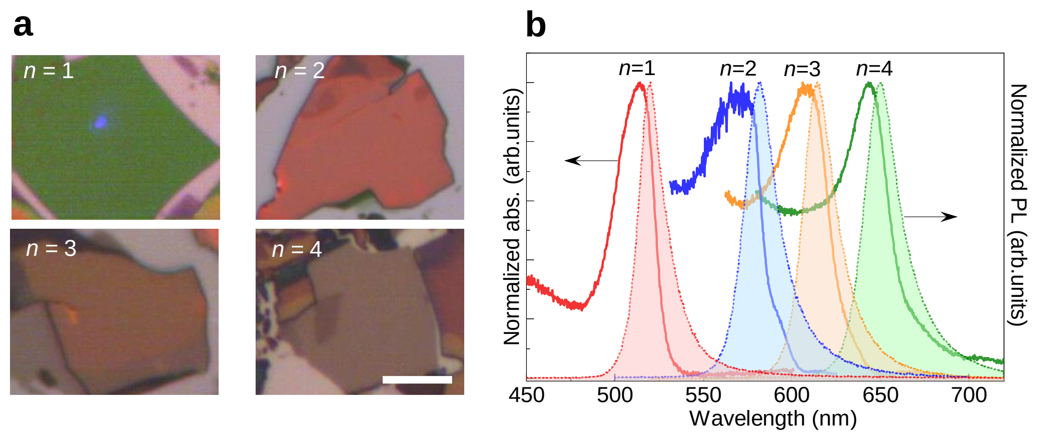

Figure S4| Absorption and emission spectra of $(BA)_2(MA)_{n-1}Pb_nI_{3n+1}$ RPP flakes. (**a**) Representative microscopic optical images of n=1-4 flakes. The scale bar is 10 µm. (**b**) Absorption and PL spectra of n = 1-4 RPP thin flakes.

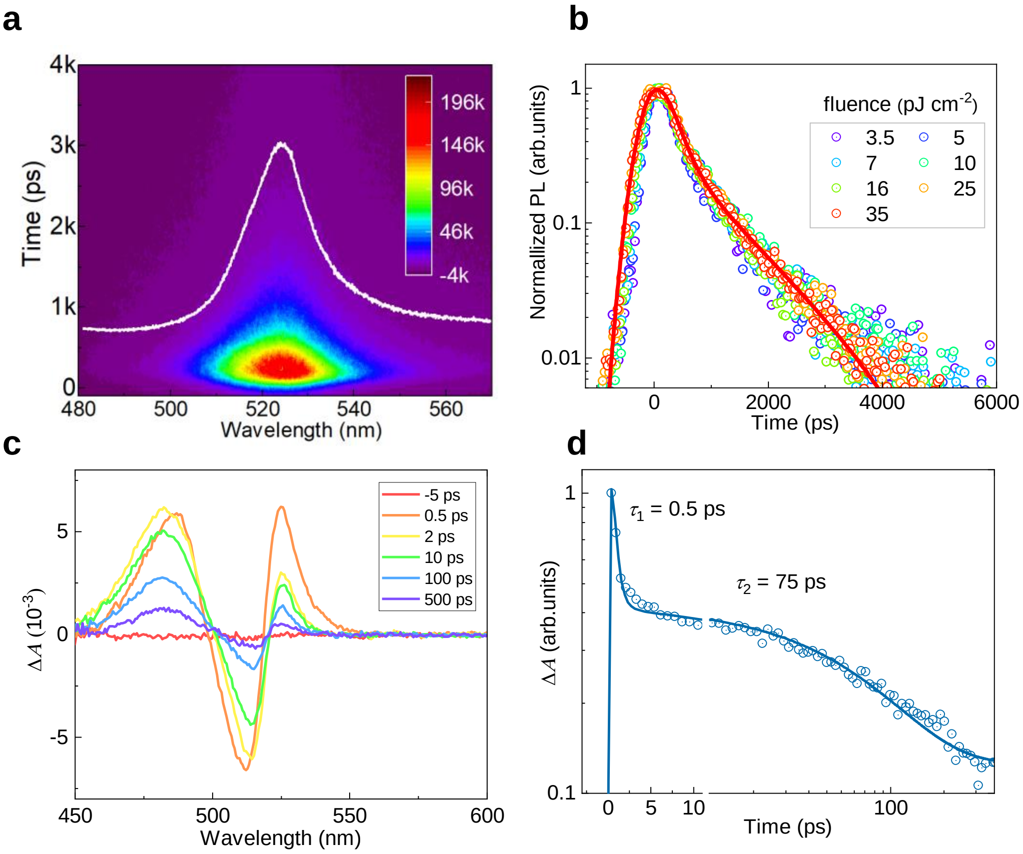

Figure S5| TRPL and TA dynamics of $(BA)_2PbI_4$ flake upon 400 nm excitation. (a) TRPL mapping. (**b**) Excitation density-dependent TRPL dynamics. (**c**) TA spectra at selected time delays (-5 ps, 0.5 ps, 2 ps, 10 ps, 100 ps, 500 ps). (**d**) TA decay dynamics. t_1~0.5 ps is attributed to the hot carrier relaxation.

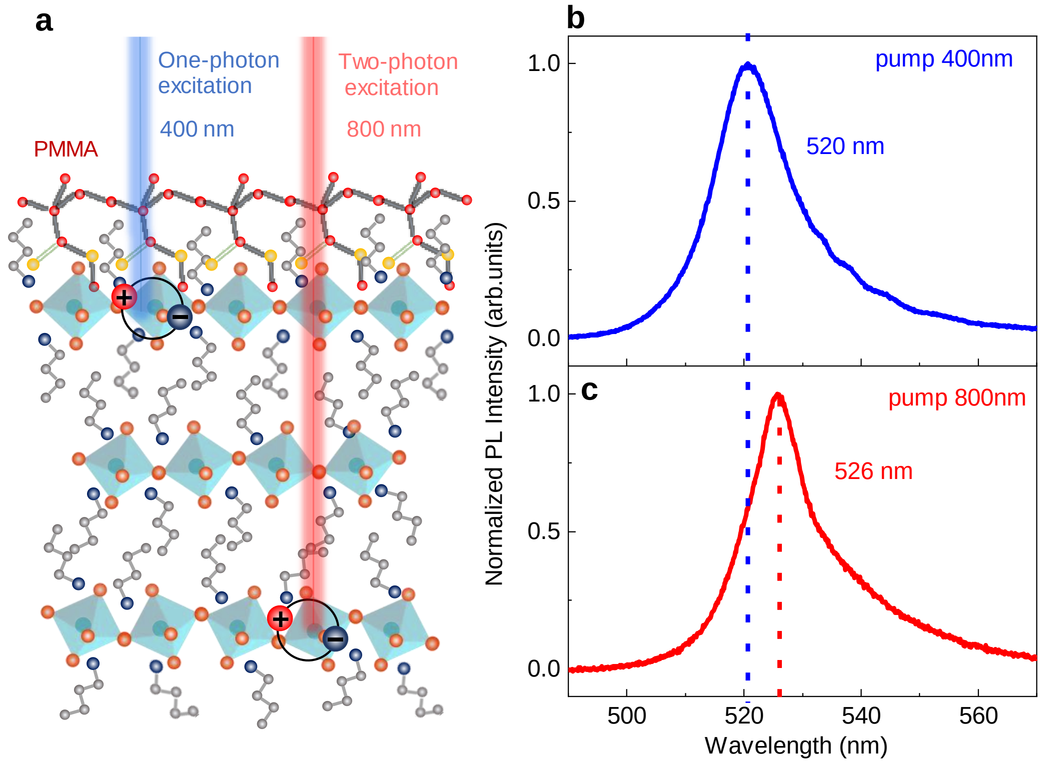

Figure S6| PL for $(BA)_2PbI_4$ flakes encapsulated with PMMA by one-photon and two-photon excitation. (**a**) Schematic diagram of one-photon (400 nm) and two-photon (800 nm) excitation fluorescence. Laser with a wavelength of 800 nm has a deeper penetration depth than 400 nm. PL for $(BA)_2PbI_4$ flakes encapsulated with PMMA by (**b**) one-photon and (**c**) two-photon excitation. We attribute PL peak at around 520 nm to the excitons generated close the surface affected by PMMA and a low energy (LE) peak at around 526 – 536 nm to the excitons generated in the bulk that is less affected by PMMA.

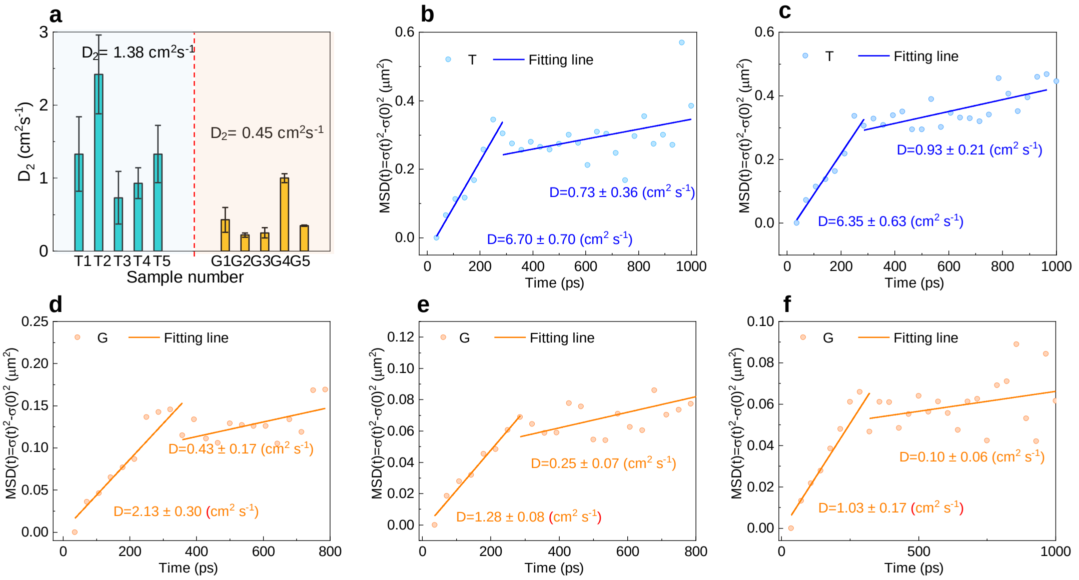

Figure S7| Slow diffusion coefficients D_2 for T and G regions. (**a**) Histogram of measured slow diffusion coefficients D_2. Representative MSD of (**b-c**) T and (**d-f**) G regions.

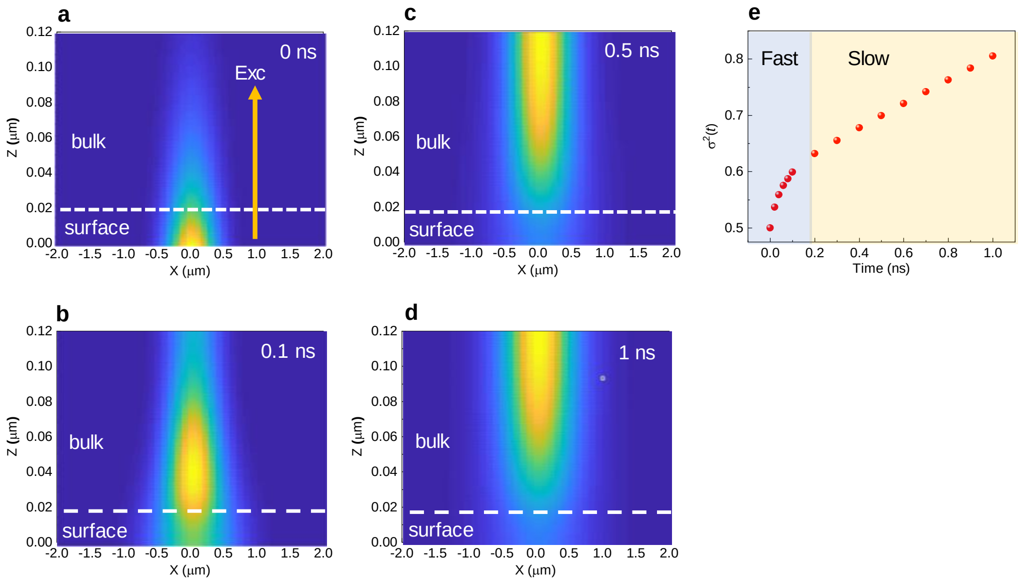

Figure S8| Theoretical simulation of the two-step diffusion. Simulated exciton distribution at (**a**) 0 ns, (**b**) 0.1 ns, (**c**) 0.5 ns and (**d**) 1 ns. (**e**) The MSD curve shows a smooth transition from the fast to slow diffusion regimes. In the simulation, the surface and bulk have a thickness of 20 nm and 100 nm, respectively. The intraplane diffusion coefficients for the surface and bulk are 8 and 0.2 cm^2s^{-1}, respectively. Their lifetimes are 100 ps and 1 ns, respectively. Both have the same interplane diffusion coefficient D = 0.06 cm^2s^{-1} adopted from the reference[1].

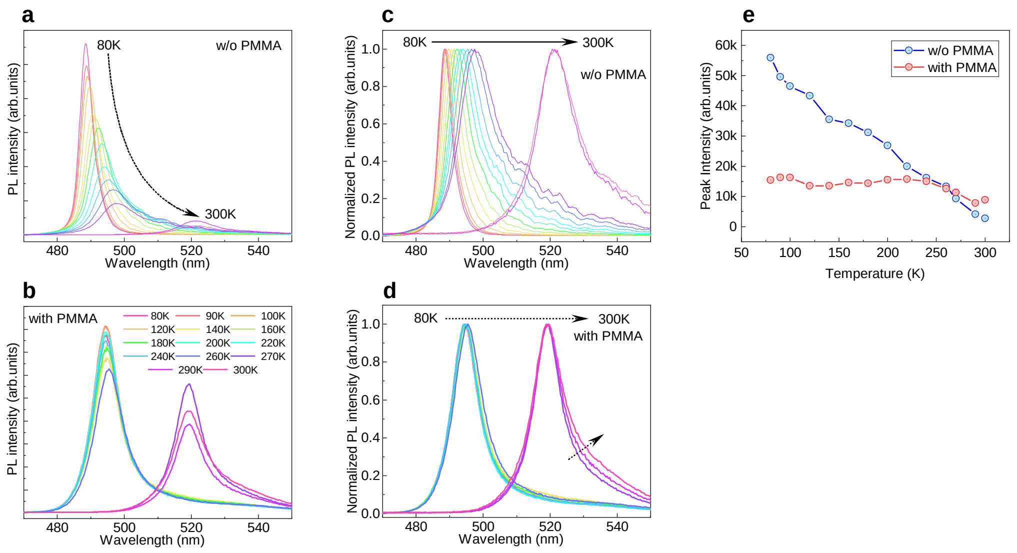

Figure S9| Temperature dependent PL for $(BA)_2PbI_4$ flakes w/o and with PMMA. Temperature dependent PL for exfoliated $(BA)_2PbI_4$ flakes (**a**) w/o and (**b**) with PMMA. Their normalized trends are shown in (**c**) for w/o and (**d**) for flakes with PMMA. (**e**) Temperature-dependent PL intensity of $(BA)_2PbI_4$ RPP flakes with and w/o PMMA.

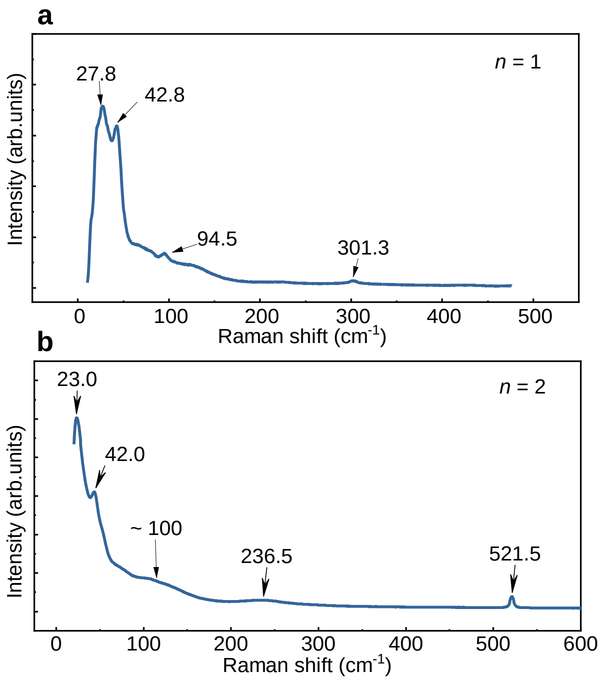

Figure S10| Raman spectra of (a) *n* = 1 RPP flake at 77 K and (b) *n* = 2 RPP flake at room temperature. The pronounced peak at 521.5 cm^{-1} (65.2 meV) is unique for BA-based RPPs and was attributed to the breathing mode of BA molecule.[2, 3]The Raman modes below 200 cm^{-1} attributed to the translations/vibrations of Pb-I framework. The peaks at 23 cm^{-1} (2.9 meV) and 44 cm^{-1} (5.5 meV) are attributed to the bending and rotation modes of the inorganic octahedral.[2]

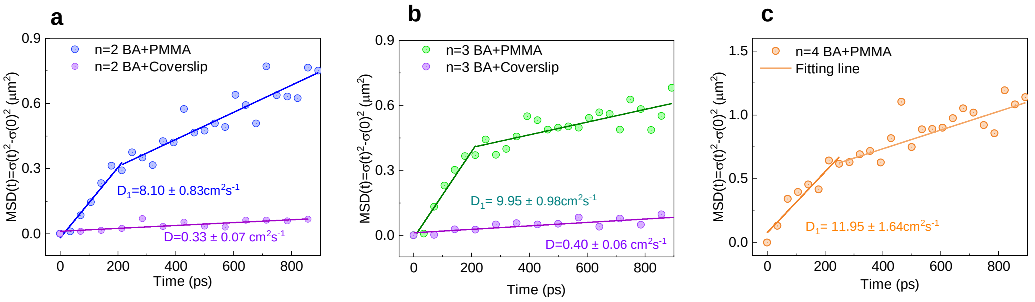

Figure S11| Mean-square-displacement of the exciton population over time of different sample in exfoliated $(BA)_2(MA)_{n-1}PbnI_{3n+1}$ RPP flakes. (**a**) *n*=2 w/ and w/o PMMA (**b**) *n*=3 w/ and w/o PMMA. (**c**) *n*=4 w/ PMMA.

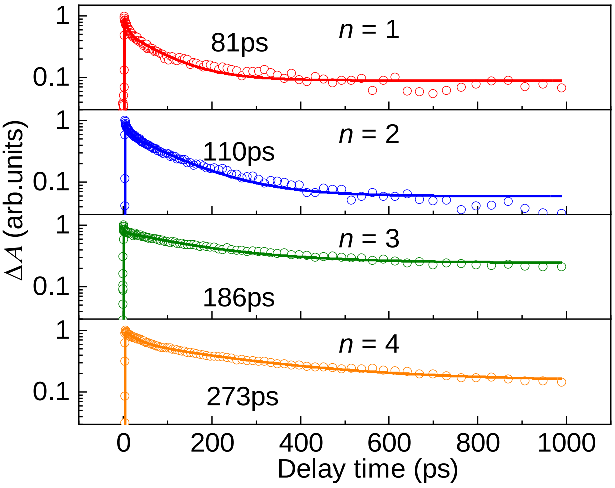

Figure S12| Transient absorption dynamics of *n* = 1-4 RPP exfoliated flakes monitored at the photo-bleaching region. Free exciton lifetime was extracted to be 81 ps, 110 ps, 186 ps and 273 ps for *n* = 1,2,3,4 RPPs.

Supplementary References :

[1] A.J. Magdaleno, M. Seitz, M. Frising, A. Herranz de la Cruz, A.I. Fernández-Domínguez, F. Prins, Materials Horizons, 8 (2021) 639-644.

[2] T. Yin, B. Liu, J. Yan, Y. Fang, M. Chen, W.K. Chong, S. Jiang, J.-L. Kuo, J. Fang, P. Liang, Journal of the American Chemical Society, 141 (2018) 1235-1241.

[3] J.M. Urban, G. Chehade, M. Dyksik, M. Menahem, A. Surrente, G. Trippé-Allard, D.K. Maude, D. Garrot, O. Yaffe, E. Deleporte, The Journal of Physical Chemistry Letters, 11 (2020) 5830-5835.
